# Clinical pregnancy rate for frozen embryo transfer with HRT: a randomized controlled pilot study comparing 1 week versus 2 weeks of oestradiol priming

**DOI:** 10.1186/s12958-023-01111-8

**Published:** 2023-07-07

**Authors:** Annalisa Racca, Samuel Santos-Ribeiro, Panagiotis Drakopoulos, Joran De Coppel, Lisbet Van Landuyt, Herman Tournaye, Christophe Blockeel

**Affiliations:** 1 Reproductive Medicine Service, Dexeus University Hospital, Barcelona, Spain; 2 IVI-RMA Lisbon, Lisbon, Portugal; 3 Department of Obstetrics and Gynecology, Faculty of Medicine, University of Lisbon, Lisbon, Portugal; 4 Centre for Reproductive Medicine, Brussels IVF, Universitair Ziekenhuis Brussel, Brussels, Belgium; 5 Department of Obstetrics, Gynecology, Perinatology and Reproduction, Institute of Professional Education, Sechenov First Moscow State Medical University of the Ministry of Health of the Russian Federation (Sechenov University), Moscow, Russia; 6 Department of Obstetrics and Gynecology, University of Zagreb-School of Medicine, Šalata 3, Zagreb, Croatia

## Abstract

**Research question:**

Does a frozen-embryo transfer in an artificially-prepared endometrium (FET-HRT) cycle yield similar clinical pregnancy rate with 7 days of oestrogen priming compared to 14 days?

**Design:**

This is a single-centre, randomized, controlled, open-label pilot study. All FET-HRT cycles were performed in a tertiary centre between October 2018 and January 2021. Overall, 160 patients were randomized, with a 1:1 allocation, into two groups of 80 patients each: group A (7 days of E2 prior to P4 supplementation) and group B (14 days of E2 prior to P4 supplementation). Both groups received single blastocyst stage embryos on the 6th day of vaginal P4 administration. The primary outcome was the feasibility of such strategy assessed as clinical pregnancy rate, secondary outcomes were biochemical pregnancy rate, miscarriage rate, live birth rate and serum hormone levels on the day of FET. Chemical pregnancy was assessed by an hCG blood test 12 days after FET and clinical pregnancy was confirmed by transvaginal ultrasound at 7 weeks.

**Results:**

The analysis included 160 patients who were randomly assigned to either group A or group B on the seventh day of their FET-HRT cycle if the measured endometrial thickness was above 6.5 mm. Following screening failures and of drop-outs, 144 patients were finally included both in group A (75 patients) or group B (69 patients). Demographic characteristics for both groups were comparable. The biochemical pregnancy rate was 42.5% and 48.8% for group A and group B, respectively (p 0.526). Regarding the clinical pregnancy rate at 7 weeks, no statistical difference was observed (36.3% vs 46.3% for group A and group B, respectively, *p* = 0.261). The secondary outcomes of the study (biochemical pregnancy, miscarriage, and live birth rate) were comparable between the two groups for IIT analysis, as well as the P4 values on the day of FET.

**Conclusions:**

In a frozen embryo transfer cycle, performed with artificial preparation of the endometrium, 7 versus 14 days of oestrogen priming are comparable, in terms of clinical pregnancy rate; the advantages of a seven-day protocol include the shorter time to pregnancy, reduced exposure to oestrogens, and more flexibility of scheduling and programming, and less probability to recruit a follicle and have a spontaneous LH surge. It is important to keep in mind that this study was designed as a pilot trial with a limited study population as such it was underpowered to determine the superiority of an intervention over another; larger-scale RCTs are warranted to confirm our preliminary results.

**Trial registration:**

Clinical trial number: NCT03930706.

## Introduction

Frozen Embryo Transfer (FET) cycles have increased ever since the first pregnancy from IVF using cryopreserved embryos was reported in 1983 [38]. While frozen-thawed embryo transfer was initially developed to perform embryo transfer in oocyte donation cycles [21], it subsequently evolved towards an elective technique for patients with supernumerary embryos and an increased risk of developing ovarian hyperstimulation syndrome [9]. Nowadays, FET cycles are also used in cases with late-follicular progesterone elevation [4, 16, 30], embryo-endometrial asynchrony [34], recurrent implantation failure [24], and pre-implantation genetic diagnosis/screening. This evolution of utility in the FET landscape also reflects itself in the currently available data for FET usage, with a 93% increase of the procedure between 2013 and 2018 [11].

A thorough look at the current FET protocols is important to gain more insight towards an optimal FET strategy. FET can take place in either a natural cycle or in an artificial cycle [23]. According to a recent Cochrane meta-analysis [14] there is no evidence to support the use of one regimen in preference to another. Nonetheless, taking into account the minimal cycle monitoring related to such practice, i.e. hormonal analyses and ultrasound scans of the endometrium, and the applicability to even women without regular bleeding, the protocol of exogenous oestrogen and progesterone administration is widely used for endometrial preparation [42]. However, this approach has some disadvantages such as costs, inconvenience, prolonged treatment (especially in case of pregnancy) and potential side-effects associated with oestrogen supplementation, i.e. increased thrombotic risk and preeclampsia [7, 39]. In fact, several observational studies have already hinted towards an increased risk of pre-eclampsia when using HRT for endometrial preparation [17, 27, 32], and a large systematic review [29] confirmed these findings with statistical significance. A relationship between the duration of oestrogen priming of the endometrium and the increased occurrence of hypertensive disorders was suggested by Roque et al. [29], while Shi et al. [35] found no differences in the occurrence of hypertensive disorders between eFET and fresh ET when eFET was performed in a natural cycle. On the other hand, oestrogen stimulation in FET-HRT activates thrombotic risk markers and a restriction in the use of unnecessary hormone exposure is important, as described recently by Dalsgaard et al*.* [8]. Moreover, still cycle cancellation due to spontaneous ovulation is an uncontrollable phenomenon that can always occur, especially when the oestrogen preparation takes long time, therefore the rationale for a shorter time to oestrogen exposure could potentially lead to less spontaneous ovulations and easier programming of the FET cycle. Contrasting results do exist, however, further emphasizing the need for additional exploration of this subject [5].

Nowadays, most FET-HRT protocols opt for the 14-day period of oestrogen supplementation to mimic the natural proliferative phase of the menstrual cycle [6]. However, scarce evidence has shown that 5 to 7 days is sufficient for endometrial proliferation [3, 26]. Recently, Sekhon et al. [33], Joly et al*.* [19] and Jiang et al*.* [18] demonstrated in retrospective cohort studies including more than thousand patients, that the length of E2 supplementation is linked neither to implantation rate, nor live birth rate and cumulative live birth rate; as well as the level of oestradiol on the day of start of progesterone [22].

Besides the very open debate about the ideal length of the E2 supplementation and considering recent results showing that this has no effects on the FET outcome, we should consider another important issue of the FET cycle, which is the delayed time to pregnancy. A recent study on patients’ perspectives regarding elective FET (eFET) revealed that the postponement of embryo transfer is an important deciding factor in the choice of eFET versus fresh embryo transfer [37]. Considering these important results, reducing time-to-pregnancy (TTP) in the FET-HRT protocol would therefore increase patient comfort when choosing eFET over fresh ET. Given the totally arbitrary decision to perform 14 days of oestrogen endometrial preparation in a FET-HRT, and the emerging evidence that the duration of oestrogen exposure does not affect success rates and given the real need to shorten time to pregnancy for patients facing IVF; the main objective of this pilot study is to evaluate the feasibility of a short endometrial preparation in FET-HRT cycles with the administration of 7 consecutive days of oestrogen priming prior to P4 initiation, by comparing clinical pregnancy rates with the standard of care (14 days of oestrogen priming).

## Materials and method

### Study population and design

This was a single-centre, randomized, controlled, open-label, pilot study. Women who were planning to undergo FET-HRT in our centre were screened and consequently invited to participate in this study. All FET-HRT cycles were performed in a tertiary referral centre (Brussels IVF, Centre for Reproductive Medicine, Universitair Ziekenhuis Brussel, Belgium) between October 2018 and January 2021, and the follow up period was 12 weeks from FET. We included all women between the age of 18 and 40 years with unexplained infertility and showing a normal uterine cavity, undergoing either IVF or ICSI with a GnRH agonist or antagonist protocol (Table 1).

**Table 1:** Inclusion/Exclusion criteria

Inclusion criteria	Exclusion criteria
▪ Women aged ≥ 18 and < 40 years ▪ Frozen embryo transfer with artificial preparation ▪ Normal uterine cavity ▪ IVF/ICSI ▪ IVF cycle with GnRH agonist or antagonist ▪ Single day 5 blastocyst transfer ▪ Top quality embryo (at least Bl 3BA) at the moment of ET ▪ Participants can be included only once in the trial	▪ Body mass index of ≤ 18 and ≥ 29 ▪ Previous diagnosis of PCOS/POI ▪ Endometriosis stage 3 and 4 ▪ Previous diagnosis of hydrosalpinx ▪ Systemic diseases such as thyroid dysfunction, unless corrected ▪ History of recurrent implantation failure or recurrent miscarriage ▪ PGT-A and PGT-M cycles ▪ Oocytes donation cycles ▪ Known abnormal karyotype of the subject or of her partner/sperm donor, as applicable, depending on the source of sperm used for insemination in this trial. In case partner sperm will be used and the sperm production is severely impaired (concentration < 1 million/mL) normal karyotype, including no Y-chromosome microdeletion, must be documented ▪ Any known clinically significant systemic disease (e.g. insulin-dependent diabetes) ▪ Active arterial or venous thromboembolism or severe thrombophlebitis, or a history of these events ▪ Current or past (within 90 days prior to screening) smoking habit of more than 10 cigarettes per day

*IVF* in vitro fertilization, *ICSI* intracytoplasmic sperm injection, *GnRH* gonadotropin releasing hormone, *ET* embryo transfer, *PCOS* polycystic ovarian syndrome, *POI* premature ovarian insufficiency, *PGT-A/M* pre implantation genetic testing for aneuploidy or monogenic disorders

Furthermore, only the first single Day 5 blastocyst transfer with an excellent quality embryo (at least Bl 3BA) was included. Women with a BMI lower than 18 or higher than 29 kg/m^2^, having a history of recurrent implantation failure/recurrent miscarriage, or showing an abnormal karyotype were excluded. Likewise, women who had a previous diagnosis of PCOS/POI, endometriosis stage 3 or 4, hydrosalpinx, or a systemic disease such as thyroid dysfunction (unless corrected) were omitted. Furthermore, PGT-A/M and oocyte donation cycles were also excluded. Written informed consent obtained from all participants of the study. The study was registered in clinicaltrials.gov with number NCT03930706.

### Study outcomes

Our primary outcome was clinical pregnancy at 7 weeks after FET while the secondary outcomes where positive hCG, assessed 12 days after FET, bioquemical pregnancy rate and miscarriage rate assessed during the first 12 weeks of pregnancy, following the definitions of the international glossary in fertility [43].

### Insemination, embryo quality assessment and cryopreservation

Fertilization was assessed 16-18 h after IVF/ICSI by the presence of two pronuclei, and further on, embryo development was evaluated daily until the cryopreservation of either cleavage-stage embryos (Day 3) or blastocysts (Day 5 and 6). Cryopreservation was performed by means of vitrification using a closed vitrification device with high-security straws (CBS-ViT-HS®; Cryobiosystems) using a combination of dimethyl sulfoxide and ethylene glycol as cryoprotectants (Irvine Scientific Freeze Kit®; Irvine Scientific). Day 3 embryos were evaluated based on the number and symmetry of their blastomeres, percentage of fragmentation, vacuolization, granulation and multinucleation. Based on all these parameters, an EQ score was assigned to all normally fertilized embryos using a predefined algorithm, which is divided into four categories: excellent, good, moderate, or poor. These four categories were used as defined by Racca et al. [28]. Fresh transfer of embryos or blastocysts was not included in this study. Blastocysts were scored according to the grading system developed by Gardner and Schoolcraft [13] based on the expansion stage, the number of cells joining compaction or blastulation, and the appearance of the trophectoderm (TE) and inner cell mass (ICM).

The following embryos were considered eligible for cryopreservation: day 3 embryos with ≥ 6 blastomeres and ≤ 50% fragmentation; day 5 and 6, fully expanded or hatching blastocysts with a type A/B/C ICM and type A/B TE. Only transfers of single day 5 embryos of excellent quality were included as part of this study. Vitrified blastocysts were evaluated (Table 1) after warming (with Irvine Scientific Thaw Kit®; Irvine Scientific). FETs of vitrified blastocyst (Days 5 and 6) were performed on the day of warming.

### Endometrial preparation, FET timing and patient randomization

**Fig. 1:**
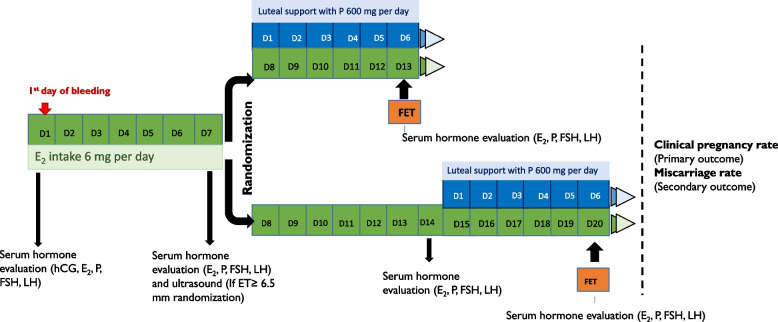
Study design flowchart. E2 (oestradiol), P (progesterone), ET (endometrial thickness), FSH (follicular stimulating hormone), LH (luteinizing hormone), FET (frozen embryo tranfer)

This study included only FETs in an artificially supplemented cycle (FET-HRT). Thus, endometrial preparation consisted of the sequential administration of oestradiol (E_2_) valerate and micronized vaginal progesterone. Patients with basal hormone values, defined as oestradiol < 80 pg/ml and progesterone < 1,5 ng/ml, and no ovarian cysts on day 1 of their cycle started administering 6 mg oral oestradiol daily. On day 7 of their treatment, serum hormone values and endometrial thickness were evaluated through a blood test and an ultrasound respectively. Patients with an endometrial thickness ≥ 6.5 mm were randomized in 2 groups with a 1:1 allocation: group A, 7 days of E_2_ priming, and group B, 14 days of E_2_ intake. Group A started 800 mg intravaginal progesterone daily (divided into 400 mg in the morning and 400 mg in the evening) on Day 8 of treatment and underwent FET on the 6^th^ day of progesterone supplementation. Group B continued 7 more days of oestradiol and started 800 mg intravaginal progesterone daily on Day 15 of E_2_ treatment and underwent FET on the 6^th^ day of progesterone supplementation. Group B received a total of 20 days of E_2_ intake before the ET, with an additional evaluation of serum hormone values and endometrial thickness on day 14 of treatment (Fig. 1).

### Assessment, data collection and randomization

Pregnancy was assessed by a blood test to evaluate hCG 12 days after FET and ongoing pregnancy was confirmed by the visualization of a fetal heartbeat during a transvaginal ultrasound at 7 weeks [31]. Data were collected in a secure and encrypted eCRF created specifically for the trial using Filemaker Pro® v13 (Filemaker Inc.) and hosted on a dedicated server at our centre (Brussels IVF). The doctors, study nurses, and research assistants collaborating in the trial were responsible for data collection.

Randomization took place on day 7 of endometrial preparation with oestradiol for all patients with endometrial thickness above 6.5 mm. The randomization was performed by means of white sealed opaque envelopes with a 1:1 allocation, and the random list was generated with sequential numbers by using STATA version 15.1 (StataCorp, College Station, Texas, USA). The study nurses’ team together with the senior clinicians involved in the study, were in charge of the enrolment, randomization and allocation.

### Sample size and statistical analysis

As there is no evidence supporting only 7 days of oestradiol priming in FET-HRT, no formal sample size calculation was performed. Therefore, we arbitrarily decided to include 160 patients.

To determine, with 80% power, superiority of one strategy over the other, considering a difference in clinical pregnancy of 10%, with a formal sample size calculation (alfa 0.05 and beta 0.2) we would have required 421 patients in each group, with a total of 842 patients.

Continuous variables were presented using mean and standard deviation while categorical characteristics as well as all primary and secondary outcomes were reported using absolute and relative values within their respective groups. Continuous variables were analysed using the Mann–Whitney U test while dichotomous variables were analysed using Fisher’s Exact test. The outcomes were reported with p values and difference of proportion. A *p*-value was considered significant whenever < 0.05. The study was conducted with respect to the Pilot study consort 2010 [10].

All statistical analyses were performed with STATA version 15.1 (StataCorp, College Station, Texas, USA).

## Results

**Fig. 2:**
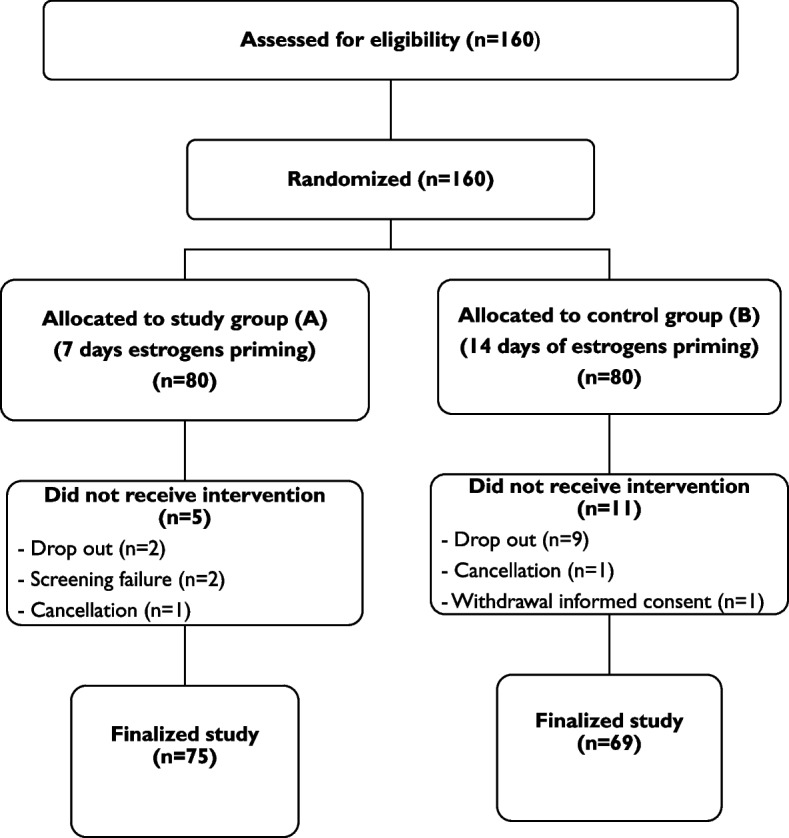
Flowchart randomization

The analysis included 160 patients who were randomly assigned to either group A or group B on the 7th day of oestradiol intake of their FET-HRT cycle. After the exclusion of drop-outs and screening failures, 144 patients were included either in group A (75 patients) or group B (69 patients) (Fig. 2).

Patients’ demographic characteristics such as age, BMI, AMH, as well as smoking habits, parity, and indication for ART, are summarised in Table 2, while cycle characteristics such as endometrial thickness on the day of P4 start as well as E2 and P4 on day of ET are summarised in Table 3.

**Table 2:** Baseline characteristics

	**Group A** **Treated 1** **(** ***N*** ** = 80)**	**Group B** **Control 0** **(** ***N*** ** = 80)**
Age (years ± SD)	33.0 ± 3.7	33.2 ± 3.6
BMI (kg/m^2^)	23.1 ± 2.9	23.9 ± 3.1
AMH (ng(mL)	2.8 ± 1.4	2.7 ± 1.9
Smoking habit N (%)		
- No	76 (95)	77 (96.3)
- Yes	4 (5)	3 (3.8)
Parity N (%)		
- 0	28 (35)	29 (36.2)
- ≥ 1	52 (65)	51 (63.8)
Indication for ART		
- Idiopathic (2)	32 (40)	40 (50)
- Tubal factor (3)	3 (3.8)	6 (7.5)
- Ovulation disorder (4)	3 (3.8)	2 (2.5)
- Male factor (1)	38 (47.5)	26 (32.5)
- Endometriosis (5)	0 (0)	3 (3.8)
- Other (6)	4 (5)	3 (3.8)
Endometrial thickness (mm)	8.5 ± 1.6	8.5 ± 1.4

Endometrial thickness was measured after 7 days of oestrogens supplementation for both groups A and B

*BMI* Body Mass Index, *AMH* anti-müllerian hormone, *ART* assisted reproductive techniques

**Table 3:** Serum hormone levels day of ET

	**Group A** **(** ***N*** ** = 80)**	**Group B** **(** ***N*** ** = 80)**	***p*** **-value**
LH	5.9 ± 4.5	7.1 ± 6.9	0.259
E2	225.4 ± 73.8	228.5 ± 100.8	0.835
P4	12.8 ± 4.8	12.8 ± 5.2	0.318

*LH* luteinizig hormone, *E2* estradiol, *P4* progesterone

Demographic characteristics for both groups were comparable. Notably, there was no significant difference in the parity of both groups. Most patients had undergone one or no previous ART cycles and had either one or no previous live births. Additionally, the indications for ART were evenly distributed between both groups. In the entire study population, the most frequent indications for ART were the idiopathic cause and male factor. There was no significant difference between E2 and P4 levels, as well as endometrial thickness measured on the day of ET.

### Primary outcome

Outcomes of the analysis are reported, following the ITT principle to avoid possible bias due to the excluded patients, (Table 4). Our primary outcome was clinical pregnancy at 7 weeks after FET, no statistical difference was found between the intervention and control group, (36.3% vs 46.3%, for group A and group B, respectively, *p* = 0.261, difference of proportions 10%, 95% CI -0.05 – 0.25).

**Table 4:** Pregnancy outcomes in both study groups (*N* = 160)

	**Group A** **(** ***N*** ** = 80)**	**Group B** **(** ***N*** ** = 80)**	***p*** **-value**	**Proportion difference**	**95%C.I**
Pregnancy rates	34/80 (42.5)	39/80 (48.8)	0.526	0.063	-0.09- 0.21
Biochemical pregnancy	5/34 (14.7)	2/39 (5.1)	0.443	-0.096	-0.23 – 0.04
Clinical Pregnancy rate	29/80 (36.3)	37/80 (46.3)	0.261	0.1	-0.05 – 0.25
Miscarriage rate	4/29 (13.8)	3/37 (8.1)	0.690	-0.06	-0.21 – 0.096
Live birth rate	25/80 (31.3)	34/80 (42.5)	0.190	0.11	-0.03 – 0.26

### Secondary outcomes

Positive pregnancy rate was 42.5% and 48.8% for group A and group B, respectively (p 0.526, difference of proportions 0.6, 95% CI -0.09 – 0.21). Biochemical pregnancy rate was 14.7 versus 5.1 for group A and B, respectively (*p* = 0.443, difference of proportions -0.096, 95% CI -0.23 – 0.04) and miscarriage rate was 13.8 and 8.1 for group A and B, respectively (*p* = 0.69, difference of proportions – 0.06, 95%CI -0.21 – 0–096). Live birth rate was 31.3 for group A and 42.5 for group B (*p* = 0.19, difference of proportions 0.11, 95% CI -0.03 – 0.26).

### Serum hormonal levels on day of ET

The oestradiol levels on the day of ET were 225.4 and 228.5, respectively (*p* = 0.835) while the levels of P4 were comparable between the two groups (12.8 ng/ml mean for both arms, p value 0.318). LH levels also were similar with 5.9 and 7.1 for groups A and B, respectively (*p* = 0.259).

## Discussion

To the best of our knowledge, this is the first randomized controlled study investigating a shorter endometrial exposure to oestrogens (only 7 days) before starting P4 supplementation in a FET with HRT. In fact, the current results show similar clinical pregnancy rates between 7 versus 14 days of oestrogens priming in a FET HRT, opening the floor for future larger scale RCTs with the aim to confirm these exploratory results.

The wider implications of such a study are the TTP reduction which could reduce the costs but also increased patient comfort and acceptance when choosing FET over fresh ET [37], and the safety. In fact, even in terms of safety, a shorter duration of E2 supplementation could provide an advantage relating to the possible reduced risk of thrombotic and hypertensive disorders associated with the FET-HRT protocol [8].

The results of the present study are in line with a retrospective cohort study that was published in 2022, [18], in which 4142 FET-HRT cycles were divided according to 7 vs 14 days of oestrogen exposure and found no difference in cumulative live birth rate.

The basis of our hypothesis to shorten the TTP was inspired by Navot et al*.* [25] reporting that it is biologically feasible to simulate the essential hormonal and endometrial milieu of a fertile menstrual cycle and early gestation solely by the administration of oestrogen and progesterone. Since then, the length of oestrogen supplementation has been empirically chosen as 14 days to mimic the follicular phase of a physiological menstrual cycle. However, it is generally acceptable that P levels are the driving force behind endometrial receptivity [12, 20, 36]. Thus, we did not expect different cycle outcomes when using good quality blastocyst and the ET was done with a protocol in which solely the duration of E2 priming varied.

The duration of oestrogen administration before frozen embryo transfer did not impact implantation nor clinical pregnancy and early pregnancy loss or live birth rate from a statistical point of view, as shown by Sekhon et al*.* [33] and Joly et al. [19]. However, based on their results, the mean length of oestrogen supplementation was 17 and 20 days, respectively. The results of the present study are in line with Sekhon and Joly and co-authors, demonstrating no difference in outcomes between the two groups, however, it is important to acknowledge that we compared shorter time of exposure to oestrogens. Furthermore, we should also point out that, although statistically irrelevant, the difference in clinical pregnancy between to two groups was about 10% in favour of the 14 days (group B), therefore we warrant caution in opting for such a short protocol, until larger scale studies will confirm, or not, our results.

Our primary and secondary outcomes were similar to the results of the FET-HRT population in most studies comparing eFET with fresh ET [1, 2, 40]. The oestradiol values on the day of ET were comparable between the two groups, although a significant difference in the time of exposure. The hormonal results on the day of ET were in line with a previous study from our group where it was reported by Mackens et al. [22] that oestradiol levels do not influence the outcome of the FET-HRT cycle.

### Limitations and strengths

Considering the lack of evidence related to a shorter exposure to oestrogen in an HRT cycle, we did not have enough knowledge and evidence to directly perform a powered RCT. Therefore, a major limitation of the present study is the design as a pilot trial. As a result of its limited study population, it was underpowered to determine the superiority of one intervention over another. Instead, the purposes of the present study were to explore trends in pregnancy rates for each HRT strategy and to provide us with enough knowledge for the sample size calculation of further definitive RCTs in which a non-inferiority of the 7 days E2 priming approach could be confirmed. Although the pilot design, these results allow us to safely design a larger confirmatory RCT, exploring the safety and efficacy of a FET-HRT with the aim to reduce TTP.

A vital strength of this trial is its strict inclusion criteria, such as the choice to include only single transfer of good quality blastocysts [15, 41]. This allowed us to truthfully compare the influence of reduced time of oestrogen priming on cycle outcomes. Furthermore, the study was conducted in a rigorous way with respect to the Pilot study consort 2010 [10].

Lastly, we performed a hormonal assessment on the day of ET to understand the possible influence of the different protocols on hormonal trends and cycle outcomes, to have a fully comprehensible understanding of the HRT protocol.

## Conclusions

In a frozen embryo transfer cycle, performed with artificial preparation of the endometrium, 7 versus 14 days of oestrogen priming are comparable, in terms of clinical pregnancy rate; the advantages of a seven-day protocol include the shorter time to pregnancy, reduced exposure to oestrogens, and more flexibility of scheduling and programming, as we could program with 7–8-9 days of oestrogens intake and yield similar results, even with less probability to recruit a follicle and have a spontaneous LH surge. These are the main results of the present pilot-controlled trial, which needs to be confirmed firstly with endometrial outcomes measures, such as molecular expression and, secondly, with future larger-scale RCTs.

## Data Availability

Full data set is available from the corresponding author, upon reasonable request.
